# Cadaveric Analysis of the Kambin's Triangle

**DOI:** 10.7759/cureus.475

**Published:** 2016-02-02

**Authors:** Reid Hoshide, Erica Feldman, William Taylor

**Affiliations:** 1 Neurosurgery, UC San Diego

**Keywords:** kambin, triangle, minimally invasive spine surgery

## Abstract

Introduction

Kambin’s Triangle is a right triangle over the dorsolateral disc. The area of this right triangle currently serves as a strategic site of posterolateral, minimally invasive access to the nerve root for delivery of epidural steroid injections. This posterolateral approach has also been considered a safe area of access to the intervertebral disc space and, thus, an effective approach in reducing complications, such as violation of the thecal sac, the nerve root, or the bony elements of the spine during minimally invasive spinal surgery. No published studies have been performed to characterize the dimensions of the Kambin's Triangle. Our aim is to characterize its dimensions at the lumbar levels and determine its efficacy and safety as a site of access for minimally invasive spinal surgery.

Methods

Two randomly chosen adult male cadavers were used for this study. The measurements were made bilaterally at their lumbar levels (L1–L5), which equates to 16 total measurements (eight bilateral disc spaces on two cadavers). The disc space was first accessed using a Kirschner wire in a standard oblique approach. With the assistance of fluoroscopy, a Kirschner wire was passed into the disc through the Kambin’s Triangle. The procedure was performed on the cadavers bilaterally at four levels, followed by open dissection. The calculations of the area were made by measuring the exiting nerve root, the superior border of the caudal vertebra, and the superior articulating facet—the borders of the Kambin's Triangle.

Results

The Kambin’s Triangle height and width respectively averaged at 12 mm and 10 mm (L1–L2), 13 mm and 11 mm (L2–L3), 17 mm and 11 mm (L3–L4), and 18 mm and 12 mm (L4–L5). Thus, the area at each level was 60 mm^2^ (L1–L2), 71.5 mm^2^ (L2–L3), 93.5 mm^2^ (L3–L4), and 108 mm^2^ (L4–L5). All dissected levels demonstrated adequate anchoring of the Kirschner wire into the disc space with no evidence of nerve injury. Following this, a retractor was placed and complete discectomies were performed. All exiting nerves were protected in this safe zone and the thecal sac remained inviolate.

Conclusion

Understanding the Kambin’s Triangle will assist surgeons in the minimally invasive approach to spinal surgeries, with potentially safe placement of interbody cages through this strategic space.

## Introduction

**Figure 1:**
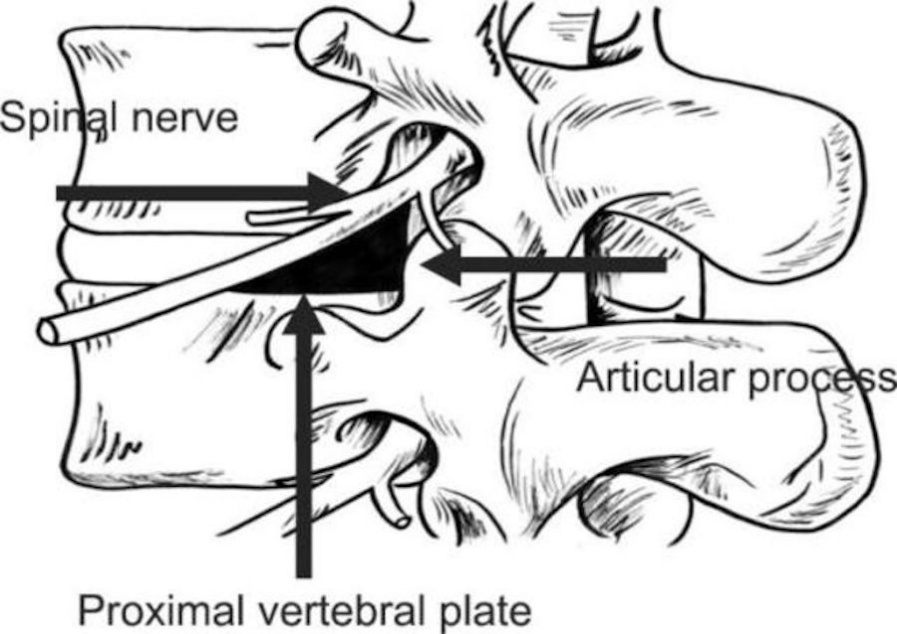
The anatomic perimeters of the Kambin’s Triangle.

**Figure 2:**
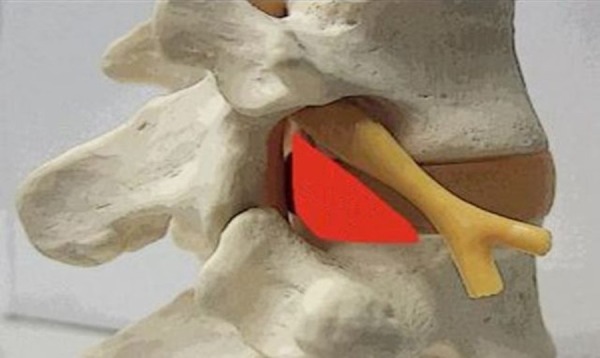
An anatomic model of the Kambin’s Triangle (Kambin’s Triangle outlined in red).

The Kambin’s triangle is a three-dimensional anatomic right triangle over the dorsolateral intervertebral disc of the lumbar spine [1]. In a two‑dimensional plane, the boundaries of the Kambin’s triangle are the superior endplate of the inferior vertebral body (base of the triangle), the superior articulating facet (the height of the triangle), and the exiting superior nerve root (the hypotenuse of the triangle), as depicted in Figures 1-2.

This landmark has been described many times in medical literature in the context of fluoroscopic access to the epidural space. Access to this space has been utilized for purposes of minimally invasive steroid injections, which aim directly at the nerve root of interest [1-5]. The area within this right triangle may also serve as a safe site for convenient access to the intervertebral disc space [6]. Within this space, there are no traversing neural, vascular, or visceral structures of critical importance. A previous retrospective study had demonstrated the presence of the artery of Adamkiewicz traversing within this triangle, although most occurred above the level of L3 (92%) [7]. This site of intervertebral disc access has been used for radiographic purposes in discograms [8], pain management purposes for steroid injections [4-5], and more recently, for percutaneous minimally invasive surgical approaches to intervertebral disc for discectomies and spinal fusions [6, 9]. This retroperitoneal, posterolateral approach to the lumbar spine has been considered a safe area of access to the disc space and, thus, an effective approach in reducing complications, such as violation of the thecal sac, injuries to the nerve root, or destabilization of the bony elements of the spine. Moreover, its minimally invasive nature can avoid the complications and morbidity associated with the anterior or posterior approaches to the same disc space. No published studies have been performed that quantify the dimensions and area of the Kambin's triangle. The aims of this study are to determine if fluoroscopic access to the disc space with a Kirschner wire could be performed safely, to quantify the dimensions and area of the Kambin’s triangle, and to determine the feasibility and safety of performing a discectomy through the borders defined by the Kambin’s triangle using conventional spinal surgical equipment.

## Materials and methods

Two randomly chosen adult male human cadavers were used in this study. This cadaveric study lacked personal identifiers, and no research was performed on their living relatives; therefore, Institutional Review Board (IRB) approval was not necessary. Each subject was placed in the prone position. We aimed to access and measure the dimensions of the Kambin’s triangle at all lumbar intervertebral disc levels (L1–L2, L2–L3, L3–L4, and L4–L5) bilaterally. This quantified a total of four measured values for each side (four total lumbar intervertebral discs), eight for each cadaver, and, thus, a total 16 for the purposes of this study. The L5–S1 disc space was not chosen as a feasible disc space to access because the iliac crests made lateral access difficult. Lateral fluoroscopic images were obtained, such that the disc space was appropriately aligned for direct 90-degree visualization between the vertebral body endplates and the horizontal plane. The facet joints were fluoroscopically aligned in a similar fashion, allowing both left and right superior articulating facet joints to superimpose each other, indicating its proper oblique alignment. Once fluoroscopically optimized, a Kirschner wire was advanced percutaneously through retroperitoneal space and docked into the dorsal intervertebral disc space following a stab incision of the skin. Because the traversing nerve root lacks radiopacity, only two of the three dimensions of the Kambin’s Triangle could be appreciated fluoroscopically. If aligned correctly, the traversing nerve root should split the intervertebral disc space equally between the dorsal and ventral halves. Thus, the dorsal half of the intervertebral disc space was accessed while being mindful of the dorsal border of the Kambin’s triangle, the superior articulating facet joint. The procedure was performed on both cadavers bilaterally at each of the four levels (L1–L5), followed by open dissection. Once open, the travel of the Kirschner wire was thoroughly inspected to assess any injuries made by the placement of the wire. Following this, the Kirschner wires were removed and measurements were made bilaterally at each lumbar level (L1–L5) and recorded. We had a total of 16 measurements (four bilateral disc spaces on two cadavers). Calculations of the area were made by directly measuring the length of the exiting nerve root, the length of the superior border of the caudal vertebra, and the height of superior articulating facet—the borders of the Kambin's Triangle. The area of the triangle was calculated by the geometric formula: area = 0.5 (base x height). The height, for the purposes of this study, was the length of the superior articulating facet from the superior endplate of the inferior vertebral body to the point where the traversing nerve root intersects with this imaginary vertical line. The length of the base was measured by the superior endplate of the vertebral body, bordered by the intersection of the traversing nerve root ventrally to the margin made by the superior articulating facet joint dorsally.

Following the recording of these measurements, discectomies were performed through the dissection corridor and through the landmarks defined by the Kambin’s Triangle with conventional spinal surgical instruments. The discectomies were performed on the left side for the first cadaver and on the right side for the second cadaver. Following each discectomy, the thecal sac, nerve root, and the vertebral body endplates were thoroughly inspected for any evidence of physical injury related to the discectomy process.

## Results

**Figure 3:**
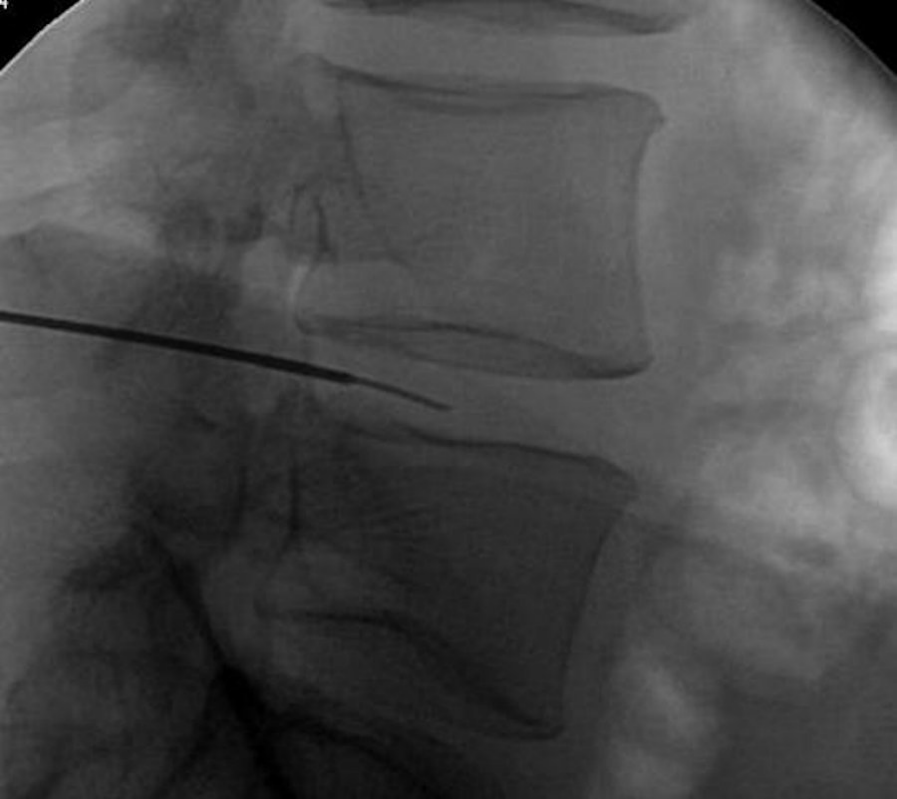
Fluoroscopic-assisted advancement of the Kirschner wire through the Kambin’s Triangle (Direct lateral projection).

**Figure 4:**
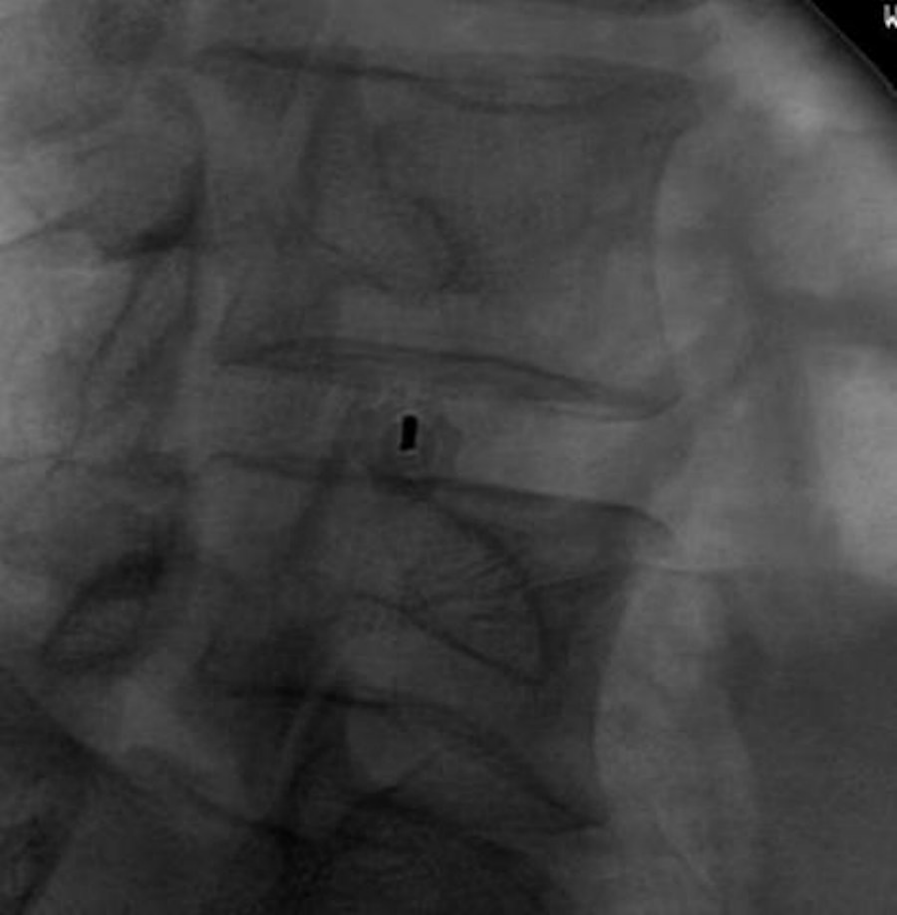
Fluoroscopic-assisted advancement of the Kirschner wire through the Kambin’s Triangle (Oblique projection).

The access of the lumbar intervertebral disc space through the Kambin’s triangle was performed as described above on two cadaveric specimens without complication. The Kirschner wires were fluoroscopically placed in the disc space without event, as shown in Figures 3-4.

Following fluoroscopic assistance of the Kirschner wires at each lumbar intervertebral disc space level bilaterally, open dissection was carried out to obtain measurements of the Kambin’s Triangle, and to visually inspect the safe placement of the previously placed Kirschner wires. All open-dissected levels demonstrated adequate anchoring of the Kirschner wire into the disc space without evidence of neural, thecal sac, or bony violation. The measurements of the Kambin’s Triangle were carried out following the visual inspection of the Kirschner wires. The base and height, respectively, averaged between each specimen bilaterally were 12 mm and 10 mm (L1–L2), 13 mm and 11 mm (L2–L3), 17 mm and 11 mm (L3–L4), and 18 mm and 12 mm (L4–L5). Thus, the area at each level averaged between both specimens was 60.0 mm^2^ (L1–L2), 71.5 mm^2^ (L2–L3), 93.5 mm^2^ (L3–L4), and 108.0 mm^2^ (L4–L5), as listed in Table 1.

**Table 1:** The dimensions of the base and height of the Kambin’s Triangle, averaged between our two cadaveric specimens.

Disc space	Base (mm)	Height (mm)	Area (mm^2^)
L1–​L2	12	10	60.0
L2–​L3	13	11	71.5
L3–​L4	17	11	93.5
L4–​L5	18	12	108.0

Following this, a retractor was placed and complete discectomies were performed within this corridor of access using conventional spinal surgical equipment. Further dissection was carried out in order to perform a thorough visual inspection of the superior and inferior endplates, the exiting nerve roots, and the thecal sac. A thorough visual inspection of these elements revealed no obvious signs of injury or violation from our discectomy procedure or the Kirschner wire access. The artery of Adamkiewicz was not appreciated in any of our cadaveric specimens of the lumbar spine.

## Discussion

Familiarity with the Kambin’s triangle will empower spine surgeons to confidently perform minimally invasive access to the intervertebral disc space of the lumbar spine. Moreover, detailed understanding of this strategic triangle can provide for precise, safe discectomies and the potential placement of interbody fusions. Alternatives to the posterolateral approach to the intervertebral disc space include anterior and posterior approaches, each of which carries its own risks and rewards. Anterior approaches can be fraught with visceral injuries to the bowel, ureters, vasculature, descending nerves, and abdominal wall [10-11]. The more common concern is the risk of retrograde ejaculation by way of injury to the superior hypogastric plexus, which courses in proximity to the anterior lumbar spine [11]. Careful patient selection must also be undertaken for an anterior approach. Spine surgeons will sometimes consult a general or vascular surgeon for the approach, where an ideal body habitus and careful vascular anatomic analysis of the aorta and inferior vena cava will need to be ascertained to determine a safe and feasible access to the anterior lumbar spine [12]. If performed correctly, injuries to these structures can be avoided through this posterolateral technique. Anterior approaches require an incision of the anterior longitudinal ligament to access the lumbar disc space, which is not a necessary step in posterolateral access surgery. Posterior approaches to the spine may risk injury to the thecal sac via excessive lateral-to-medial retraction of the thecal sac in order to obtain access to the disc space [13-14]. Posterior approaches also require an incision of the posterior longitudinal ligament and dissection of the erector spinae muscles [13]. Because the posterior bony and muscular structures must be dissected off to access the disc space, this is considered to be a destabilization procedure that requires stabilization by subsequent instrumentation [13-14]. These risks are minimized through the posterolateral approach through the Kambin’s triangle. There has been a study that raised the concern of the artery of Adamkiewicz [7] potentially running through this safe zone, which requires the cognizance of the physician or surgeon accessing this area. Knowledge of the dimensions and the area of this triangle can provide confidence to spine surgeons that they have as much information as possible to perform minimally invasive discectomies and fusions safely.

## Conclusions

Our cadaveric study analyzed the dimensions and safety of the Kambin's triangle. Through our surgical techniques and dissections, we confirmed that the Kambin's triangle is a feasible landmark for accessing the lumbar intervertebral disc. Knowledge of the dimensions and the area of this triangle can provide confidence to spine surgeons that they have as much information as possible to perform minimally invasive discectomies and fusions safely.
